# Building an urban ‘renaissance’: fragmented services and the production of inequality in Greater Downtown Detroit

**DOI:** 10.1007/s10901-015-9483-0

**Published:** 2016-01-05

**Authors:** Brian Doucet, Edske Smit

**Affiliations:** 1 Department of Social and Behavioral Science, Erasmus University College, Nieuwemarkt 1A, 3011 HP Rotterdam, The Netherlands; 2 Graduate of the Research Master in Human Geography and Planning, Utrecht University, Utrecht, The Netherlands

**Keywords:** Detroit, Urban renaissance, Shrinking cities, Municipal services, Fragmentation

## Abstract

Downtown Detroit has been undergoing a renaissance in recent years which is in stark contrast to the economic and social situation in much of the rest of the city. This renaissance has been taking place despite the city’s ability to provide good municipal services such as streetlights, security, public space and transport. This article focuses on how four areas which constitute part of Greater Downtown Detroit have relied on different combinations of actors to create and provide the services and amenities deemed necessary for capital investment and middle-class consumption. Each area has its own initiatives and actors who implement them, further fragmenting the city between its core and periphery. Renewed public spaces, private police forces and resident initiatives in middle-class neighborhoods have been created to serve specific needs of the small areas they serve. Rather than being unique, Detroit is an extreme example of fragmented and polarized urbanism which is part and parcel of contemporary cities. We argue that rather than passively reflecting existing socio-spatial divides, these private initiatives in Greater Downtown Detroit actively contribute to the production of sociospatial inequalities across the city.

## Introduction

Once the national’s richest big city, Detroit is now its poorest. It is the country’s illiteracy and dropout capital, where children must leave their books at school and bring toilet paper from home (Charlie LeDuff, Detroit: an Autopsy, 2012)

With the nation’s biggest urban bankruptcy in the rearview mirror; the Motor City and the birthplace of Motown finds itself suddenly cool again. Tough, real and cheap, it has plenty of empty space to fuel any imagination. (Susan Ager, National Geographic Magazine, 2015)

The image of Detroit is rapidly changing from decline, bankruptcy, abandonment and poverty, to a reviving urban renaissance which is attracting national and international attention. Both the Charlie LeDuff and National Geographic quotes accurately describe *part* of Detroit’s recent history and *parts* of its geographic area. The timing of these quotes (2012 and 2015) represent how, in just 3 years, the leading narrative about Detroit has begun to shift from decline to renewal. Detroit’s downtown renaissance is being celebrated in the media (Austin 2014; Coyle 2014) and even in some academic circles (Florida 2013).

But equally important to the timing of these quotes are their geography. LeDuff’s quote still accurately describes much of the city of Detroit, particularly its poor neighborhoods inhabited predominantly by African Americans. The National Geographic quote captures what has been happening within approximately 7.2 square miles of the Greater Downtown area. The size and geography of this renaissance has been quantified by a group called *Detroit 7.2,* an umbrella of different businesses, foundations and other organizations concentrated in the core of the city.^1^ Within *The 7.2*, new activity, investment and growth are taking place, yet between 2000 and 2010 Detroit lost 25 % of its inhabitants. Since 2010 (when the regeneration of Greater Downtown began in earnest) Detroit’s population is estimated to have shrunk from 713,777 to 680,250^2^ and many neighborhoods remain in freefall (Galster 2012).

This context—a reviving and prospering core and increased poverty and disinvestment in the periphery—while extreme in Detroit, is characteristic of contemporary urbanism. The focus of our paper is on how this impacts the provision of services and amenities, specifically in the revitalizing parts of a shrinking city. As Kinder (2014, p. 1768) notes: ‘The twentieth century ideological aspiration of centralized municipal governments providing universal services has faded. A neoliberal rhetoric of government austerity and market-based provisioning has taken its place.’ This has led to increased fragmentation, privatization and speculative flagship developments (Harvey 1989). In such a context, the type, access to and nature of services, amenities and provisions reflects the core-periphery concept of Graham and Marvin (2001), with more prosperous and central areas benefiting from good quality services and amenities (often provided by private initiatives) and economically and spatially peripheral areas having to rely on poor quality municipal services.

The aim of our article is twofold. First, we will examine how different actors and stakeholders in four areas that constitute part of *The 7.2* work towards providing services and amenities which are otherwise lacking. Given the extreme financial situation of the City of Detroit, culminating in its 2013 bankruptcy, many of the key services requisite for economic growth, inward capital flows and gentrification are absent, leaving a combination of businesses, institutions, foundations and middle-class households to fill this void. Second, we will critically examine the types of amenities and services which have been produced and what they mean for the city as a whole. The way in which services and spaces are being reshaped and privatized within *The 7.2* raises important questions about who can access the economic, social and safety benefits of Detroit current ‘renaissance.’

*Detroit 7.2* comprises seven areas in the Greater Downtown. We will examine regeneration and the provision of services and amenities in four of these areas. In Downtown itself, the regeneration has been spearheaded largely by wealthy businesses leaders and investors who have moved their offices to the area and now provide local amenities and services to enhance quality of life and safety. In Midtown, the area’s large public institutions, such as Wayne State University (WSU) and the Detroit Medical Center (DMC) have been primarily responsible for shaping the regeneration and spearheading gentrification. The adjacent residential neighborhood of Woodbridge has benefitted from its proximity to Midtown. Finally, the transformation of Corktown has been primarily led by a combination of residents, local businesses and volunteers.

The remainder of the paper is structured as follows. Section two will provide the theoretical and contextual overview of governance in declining cities and their neighborhoods. Section three will introduce the research methods. Section four will provide background on Detroit. Section five will offer analyses of the four areas studied. Finally, the last section will examine how the initiatives described in the paper lead to the production of inequality across Detroit.

## The consequences of population and economic decline in cities

Shrinking cities exist in a context where much of the city is unattractive for capital to invest (Herscher 2012) and there is an otherwise unfavorable set of conditions for regeneration to occur (Baldassare 1982; Pacione 2009, p. 215). This is not to say that capital is not present in these cities; Akers (2013) demonstrated, capital is indeed active in declining cities, but it is not productive. However, investment is needed to stimulate growth and development. But as Graham and Marvin (2001) demonstrate, such capital flows exist at certain sites within cities and lead to fragmented urbanism.

At a city level, shrinking cities experience a number of problems as the result of population and economic decline. This can include the inability to pay for, or support basic services and amenities that residents, businesses and visitors expect in a city. Consequences can also include poorly maintained infrastructure, declining quantity and quality of city services, decline of retail and social activities, lack of social dynamism and energy, increasing crime, stigmatization of the city’s public image and the loss of urban fabric and cohesion (Rybczynski and Linneman 1999; Martinez-Fernandez et al. 2012). Tax revenues are lost as people and businesses continue to leave the city. While revenues decline, public expenditures increase since the maintenance costs for infrastructure and for the upkeep of city services increase and are spread over an ever-declining population.

Neighborhoods are impacted by their city’s decline. As jobs disappear, many residents follow suit; businesses catering to them subsequently lose their clientele and close, reducing local quality of life and opportunities, which causes more residents to leave. However the poorest will be left behind as they lack the means to move (Fol 2012). Racism and discrimination may limit the relocation options for minorities (Massey and Denton 1993); this is particularly true of metropolitan areas such as Detroit, (Galster 2012; Sugrue 2005). While the neighborhood will experience a decline in population, the amount of housing initially remains constant. These abandoned houses become a threat to neighborhood stability (Glaeser and Gyourko 2005; Hollander 2011b, p. 10); they can serve as a haven for criminal activities (Kinder 2014; Accordino and Johnson 2000; Kirk and Laub 2010, p.444) or as targets for arson (Galster 2012, p. 237).

### Policy responses to shrinkage

Traditionally, there have been three types of responses to shrinking cities: growth-oriented policies, smart shrinkage and no action (Hollander 2011b, p. 11). Often no action is undertaken when urban areas begin to decline as shrinkage is often seen as a temporary phenomenon and it is therefore regarded as a taboo topic for policymakers (Pallagst 2010; Beauregard 2013). As the decline becomes more severe, the taboo around the topic decreases and a policy response usually follows. The most popular policy responses to shrinkage are growth-oriented strategies (Hollander 2011b, p. 11). Motivated by a growth discourse (Logan and Molotch 2007) and the shift from urban managerialism to urban entrepreneurialism (Harvey 1989), local governments seek to encourage private investments with the goal of creating new employment opportunities, new housing demand and capital accumulation. Regeneration strategies often involve property-based site-specific strategies (Doucet 2009) and take the shape of gentrifying inner-city neighborhoods, flagship projects and sports stadiums. The focus of these strategies is to upgrade local quality of life in order to attract and retain investments and wealthier, middle class households (Florida 2002; Leo and Anderson 2006). Since these strategies are mostly focused on an outside audience, they therefore may reinforce social, economic, cultural and spatial divisions within the city (Schatz 2008; Fol 2012). At a city or regional level, they are unlikely to reverse the decline since larger structural forces (globalization, deindustrialization, suburbanization and employment decentralization) undermine these local efforts (Hollander 2011b, p. 11). Research shows that they tend to give a poor return on public investment (Rybczynski and Linneman 1999; Harvey 2000), and require constant financial injections for maintenance and upkeep, what Harvey calls feeding the ‘Downtown Monster.’

According to many scholars, smart shrinkage strategies may be more advisable than growth-oriented policy responses (Rybczynski and Linneman 1999; Schilling and Logan 2008; Hollander 2011a). But this requires a different planning paradigm (Oswalt 2006; Pallagst 2010; Audirac et al. 2010). Regeneration strategies must address physical, economic, environmental and social concerns in a comprehensive way (Schatz 2008). They must aim to regenerate and empower the local community and stabilize dysfunctional markets (Schilling and Logan 2008). These regeneration strategies should also focus on increasing quality of life for poorer residents but that requires financial resources which shrinking cities do not have (Fol 2012).

### Neighborhood and area regeneration in a shrinking city

It is not just city-wide interventions which are important; regeneration efforts at the local level matter (Fol 2012) as they can make neighborhoods more livable. In order for neighborhoods to regenerate they need to alleviate key quality of life problems. (Baldassare 1982; Pacione 2009, p. 215). In many cases, vast amounts of resources and management are required, which involves the inclusion of powerful agents such as strong residents groups, community organizations, the private- and charitable-sectors and governments (Carmon 1999, p. 155; Metzger 2000). The composition of these actors is a product of the specific locations involved and will subsequently affect the regeneration type and scale (Fol 2012).

The capability of public–private partnerships or other localized groups to regenerate neighborhoods is uneven and may lead to ‘islands of renewal in seas of decay’ (Berry 1985). David Harvey’s (2000) work on Baltimore examined the role which the Inner Harbor flagship project and downtown redevelopment played in furthering inequalities across the city. Some more stable neighborhoods with a strong community, or high social capital, manage to become safer and better places as the local community organizes itself (Temkin and Rohe 1996, 1998). However, many of these areas are middle-class enclaves. Within bottom-up solutions at a neighborhood level, a distinct geography emerges, with stronger and more organized communities able to tap into economic resources and networks more so than poorer and unorganized ones.

In cities or neighborhoods where the local government is unable to provide services and amenities to improve quality of life and larger partners are also lacking, residents must take on these roles for themselves. Kinder’s (2014) work on do-it-yourself, or ‘guerilla urbanism’ examined how local residents took the initiative to try to stabilize their low-income neighborhoods. She found five particularly prominent strategies local residents in southwest Detroit employed in order to prevent abandoned houses from falling prey to scavengers, drug dealers or other unwanted activities: staging abandoned houses to look occupied, marking empty homes as claimed and protected spaces, enlivening them to show community strength, making empty houses more difficult to enter and sabotaging abandoned buildings through deliberately setting fire to them. While such DIY urbanism has been studied in lower-income neighborhoods, our focus is examining how core parts of the city use similar ideas for providing their own services within the context of a city which is unable to provide it for them.

## Detroit: an extreme example of urban decline

In the mid-Twentieth Century, Detroit was an industrial powerhouse and a place where one could pursue the ‘American Dream.’ Its decline began shortly after reaching its peak population of over 1.8 million people in 1950 (for detailed accounts of Detroit’s decline, see Galster 2012; Sugrue 2005). Deindustrialization and globalization impacted Detroit especially hard due to the city’s (and region’s) dependence on a single industry, namely automotive manufacturing. As is often the case in shrinking cities, the poorest, often minorities have been left behind as they lack the means to move (Fol 2012). According to the US 2010 census, Detroit population is 82.7 % African-American; 38.1 % individuals live below poverty level and Detroit’s median household income is $26,955 (Census 2010). In 2010, Detroit’s population was 713,777 and this decline has continued. A 2012 survey suggested that 40 % of the residents would leave in 5 years if they could due to fear of crime and lack of services (McDonald 2012; Doucet 2013). On 18 July 2013, Detroit filed for Chapter 9 bankruptcy protection, having $18 billion in debt and long-term liabilities.

Historically, Detroit’s response to this decline has been met with a variety of growth-oriented strategies, which largely came in the shape of site-specific, property-based developments aimed at stimulating inward investment or increasing tourism. Two early responses were the Renaissance Center (1970) and the GM Hamtramck Assembly Plant (known colloquially as the ‘Poletown Plant’). This massive project on the east side of Detroit involved using eminent domain to buy up an entire neighborhood which was demolished, displacing over 4,000 people (Wylie 1990; Glaeser 2011). In its place was the construction of a new auto factory. Other urban entrepreneurial responses have been concentrated Downtown and include the construction of entertainment venues such as the Joe Louis Arena (1970), the Tigers’ baseball stadium Comerica Park, Ford Field and three downtown casinos. In 1987, the city opened the Detroit People Mover, a three mile elevated rail system that requires a subsidy of 8.5 million dollars per year (Eisinger 2000; McCarthy 2002; Glaeser 2011, p. 62).

Today such growth-oriented approaches can still be seen, particularly in Greater Downtown Detroit. The city is supporting the construction of a new hockey arena for the Red Wings which will replace the Joe Louis Arena and a new 3.3 mile light rail line is currently being constructed along Woodward Avenue.

The planning paradigms in Detroit have already shifted towards planning for decline, rather than growth. The *Detroit Future City* plan accepts that the population will not grow and may continue to shrink in the future. As Kinder (2014, p. 1770) succinctly states: ‘Instead of looking towards infrastructural expansion, the primary challenge in Detroit was to manage the decay of infrastructure that previous owners had discarded.’ The plan, funded by large foundations, accepts this new reality: ‘Detroit’s future as a city [sic] will not regain its peak population of nearly 2 million people’ (DFC 2012, p. 5). It focuses efforts on Detroit’s core centers of employment and population, aiming to concentrate residents, jobs and activities in these areas. Even before the *Detroit Future City* plan, some of Detroit’s services and amenities were transferred out of the City’s control. John Gallagher (2013) notes the case of the Eastern Market, which was privatized and taken over by the foundation-funded Eastern Market Corporation in 2006. To Gallagher, the improvements brought about by this change in governance represent a positive way in which city government can be reinvented and reimagined. He notes that: ‘it illustrates so well how *pieces* of municipal government can work so much better if spun off into special-purpose entities—conservancies, authorities, quasi-public corporations and the like’ (p. 49). The city’s RiverWalk and Campus Martius (see below) are two other examples of the reinvention of governance in Detroit.

## Methodology

Our research took place in the context of a city where many services and amenities no longer remained under direct municipal control. We examine four neighborhoods in Greater Downtown Detroit. Research involved a combination of desk work (analyzing policy documents, websites and newspaper and magazine articles) and interviews.

We assumed that in order for these neighborhoods to regenerate that services and amenities must be provided by actors other than the municipality. The analysis focused on who provides these services and amenities in each neighborhood. Semi-structured interviews were held with these key actors, namely residents, someone from a community development corporation (CDC) and stakeholder private sector companies (see Table 1). These actors were interviewed about how they dealt with the declining or non-existent municipal services and amenities, their involvement in the neighborhood or area and why they choose their specific neighborhood. Data collection stopped when the point of data saturation was reached. The interviews were conducted between October and December 2013.

**Table 1:** Interviews done by author

Agent	Number of interviews
New residents	5
Long-term residents	5
Community organizations (CDCs/EDS)	1
Real estate agents	2
Financial institutions	2
Other	3
Total	18

## Four regenerating neighborhoods in Detroit

**Fig. 1:**
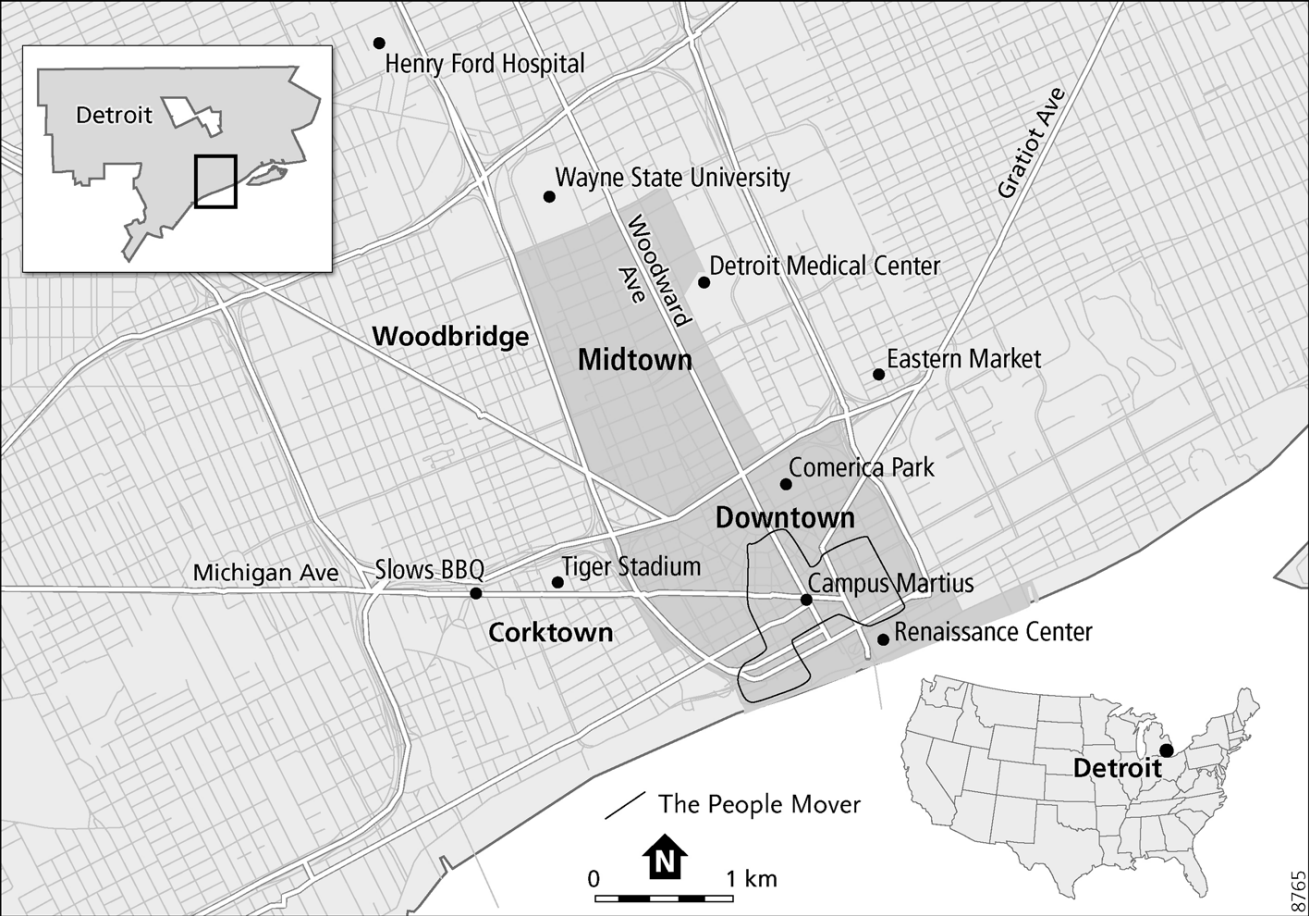
Map of Detroit

In this section we will examine each of our four case studies in turn. We will discuss what changes have occurred in each area, who the major actors or stakeholders are and how they have worked in a variety of ways in order to attempt to alleviate quality of life problems and provide services not given by the City of Detroit. Table two gives an overview of some key statistics for each area and Fig. 1 displays a map of the city with each of the four areas indicated. It should be noted that while they have all experienced some form of decline between 2000 and 2010, this is often much milder than the city as a whole.

### Downtown Detroit

Downtown is the historic heart of the city. Downtown’s fortunes were a product of the decline of the rest of the city; as Detroit’s population suburbanized, businesses catering to them also followed. Low points were when the J. L. Hudson’s department store—once the tallest in the world—closed (1983) and was later demolished (1998). Yet even as Downtown was declining, a number of speculative, growth-oriented, entertainment-led developments took place (McCarthy 2002). The most prominent of these are three casinos and two sports stadiums.

Downtown Detroit has been at the heart of Detroit’s urban entrepreneurial strategies. Organizations such as the Detroit Economic Growth Corporation, a non-profit partnership that works closely with the City of Detroit and others to support existing businesses and attract new ones to the city.

The combination of incentives aimed at attracting businesses and people to Downtown together with historically low property prices led several high-profile firms to relocate downtown, including CompuWare (2003) and Dan Gilbert’s Quicken Loans (2011). This produced some impressive statistics: Detroit was ranked second in growth in ICT companies from 1998 to 2009 (DFC 2012, p. 68). Quicken Loans itself has moved more than 15,000 of its employees Downtown and more and more workers are living in the vicinity; between 2000 and 2010 there was a 55.8 % increase in the number of households Downtown.

**Fig. 2:**
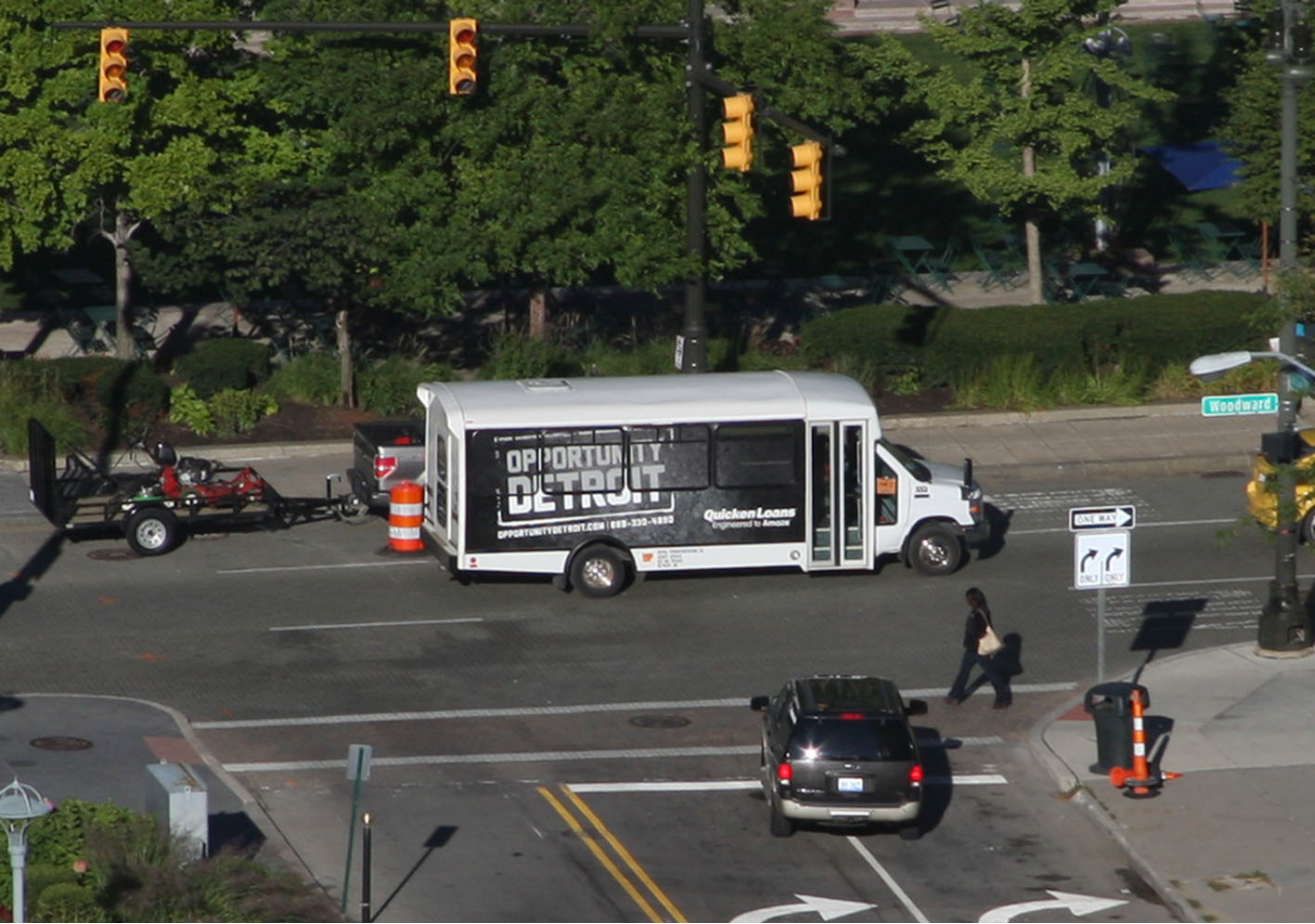
An ‘Opportunity Detroit’ shuttle bus running Downtown (Photo by: Lowell Boileau)

While Downtown has been the focal point of urban growth strategies for decades, today’s changes are largely being driven by Dan Gilbert (Akers and Leary 2014; Austin 2014; Segal 2013). The billionaire founder of Quicken Loans and a native of the Detroit-area has bought more than forty buildings Downtown, through his property arm, Rock Ventures. Opportunity Detroit, whose banners and posters can be seen all over Downtown, is part of the marketing and promotional wing of Gilbert’s growing Downtown property empire. They provide one of the amenities greatly lacking to the new Downtown employees and residents: good quality public transport. Small ‘Opportunity Detroit’ shuttle buses (Fig. 2) cater to employees working in Gilbert-owned buildings and run bus services throughout Greater Downtown. Gilbert is also one of a consortium of private-sector investors behind the construction of a new light-rail line along Woodward, from Downtown to New Center.

One of the biggest signs of gentrification in Detroit has been the growth of urban cycling. The city’s first bike sharing scheme is run by Zagster, a company specializing in custom bike share. However this scheme reflects the privatized nature of the new services and amenities which can be found in Greater Downtown Detroit. The main page on their website asks: ‘What kind of Detroit rider are you?’ This scheme is available to two types of riders: *Quicken Loans riders*—an employee of Quicken Loans or its family of companies—or *Sponsored riders*—who work for other companies which sponsor Zagster’s downtown bike share. There are ten locations to access bikes and rides are free. At the time of writing, no other bike share program was in place in Detroit, meaning this amenity remains available only to selected downtown employees.^3^

**Fig. 3:**
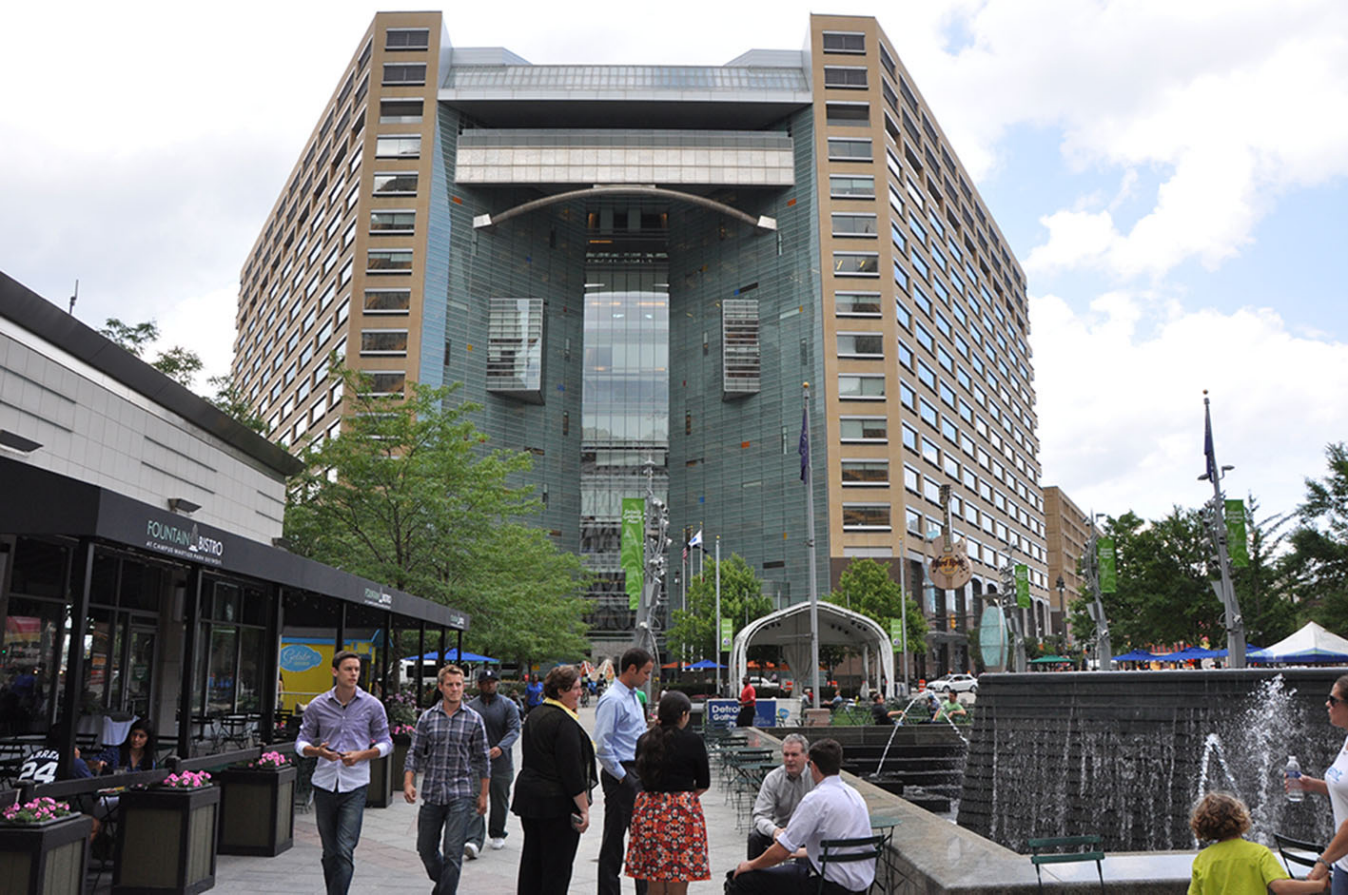
Campus Martius with the Compuware world headquarters. The building is also home to Quicken Loans

The regeneration of Campus Martius, the historic heart of Downtown, has been one of the most visible signs of change and investment. New investments into the public space have reinvented and redesigned Detroit's main civic square (Fig. 3), with significant emphasis on place making. The company hired by Gilbert to oversee the redevelopment of much of Downtown’s public spaces stated on their website:

You may have heard about downtown Detroit’s big comeback story. Campus Martius has become one of America’s great urban squares … downtown Detroit became the Rust Belt comeback kid to watch … He [Dan Gilbert] is a new kind of visionary who understands the fundamental value of great places, and the need to work with his fellow citizens to shape the city’s future together, rather than imposing a singular vision from the top down.

One of the biggest challenges in the regeneration of a city such as Detroit is dealing with crime and safety. Downtown, Dan Gilbert has provided his own solutions, as Austin’s (2012) article in *The New York Times* pointed out: “Only 35,000 of Detroit’s 88,000 streetlights actually work, so some owners are buying and installing their own. In Gilbert’s downtown, a Rock Ventures security force patrols the city center 24 h a day, monitoring 300 surveillance cameras from a control center.” (Austin 2014). This is one of the key features which have attracted new residents to live Downtown. During our interviews, a local realtor explained that people “really want to live in a tower of security and they are willing to spend a lot of money for it”. Downtown has the type of police and security that the City of Detroit – at least before emerging from bankruptcy – was unable to provide (see books such as LeDuff 2013 and Martelle 2012 for some sensational accounts of the Detroit Police Department’s lack of capabilities). In addition to the security forces, piped music and new lighting contribute to the goal of providing a safe area to work, walk around and consume (Gilbert has been influential in bringing several high profile retailers to downtown). Yet there is a cruel irony that was noted in Segal’s (2013) *New York Times* celebration of the Downtown which Dan Gilbert has created:

These guards keep an eye on what is actually one of the safest parts of the city. Few people live downtown, which means that the area is largely empty at night, and during the day it is filled with white-collar workers. There are rough patches, and a block-wide hole where the Hudson’s department store once stood. But the place feels more sterile than threatening. (Segal 2013)

The redevelopment and privatization of security around Campus Martius raises serious questions about access to public space, in this case, Detroit’s most important civic square. The policing of this space, as well as control over its design, amenities and facilities are now in private hands.

As Akers and Leary (2014) have noted, Downtown is rapidly being controlled by tycoons such as Gilbert. In many ways, Detroit has always been a product of its richest individuals. However, they note a striking difference between Gilbert and predecessors such as Henry Ford: “he [Dan Gilbert] has managed to earn a reputation for civic-mindedness mostly by attempting to lure tenants to his properties. Tycoons of an earlier era had to pursue something besides their main hustle to earn the laurels being heaped on Gilbert.”

One of the consequences of this is that while Downtown has seen new investment, residents and social activities, not everyone has benefited from this transformation. The demand for Downtown living, particularly among white, young professionals, is increasing and consequently gentrification is occurring. Rents are rising and residents are being displaced. Two of our interviewees, both African Americans who had close family members who were displaced from Downtown apartments stated that:

The rent went from 1100 to 2400 a month in a 3 to 4 year time period;

People who have been living there for 20 to 40 years have to move because of the increased rents. This has all happened in only the last two years.

The displaced now reside outside Downtown as it now lacks subsidized affordable housing. While the developments in Downtown help to bring back a tax base for the city [tax revenue from the three Downtown casinos accounted for 16 % of total city revenue in 2012 (Bromey and Gallagher 2013)], it is rapidly becoming a prosperous and safe island, economically, socially, racially pulling away from the rest of Detroit.

Many of our interviewees indicated that Detroiters were glad that improvements were taking place Downtown, and that these business tycoons could ‘accomplish things’ which the local municipality could not. As a local mortgage broker noted: “the regeneration can help to bring the tax base in place to restore our government’s functionality and then be able to fund the services that are needed for people to have a family, have a life, and have a career without having to sacrifice something that they can get in another city”. Nevertheless some question whether they would be excluded from these developments. As a long-term African American resident noted: “the focus should not be solely on Downtown because the people that revive Downtown have plenty of money and other neighborhoods should not be forgotten. Displacing the people who kept the city up and running, even though those people did not give the tax base that was needed, is not fair”. These two quotes highlight the difficulty between regenerating a declining city and maintaining spatial and social justice. While regeneration and spatial and social justice are often both a goal of the local government, this is not necessarily the case for the private sector.

### Midtown

Midtown is the largest of the four areas we studied. It includes businesses, residential areas and three of Detroit’s major anchor institutions [Wayne State University (WSU), Henry Ford Health System and Detroit Medical Center (DMC)]. These anchor institutions have been driving the current changes in Midtown. The anchor institutions cooperate to improve Midtown through a non-profit economic development organization, Midtown Inc. which has a 30 year history as the university-cultural association.

The changing relationship between Midtown Inc and the neighborhood around it has been central to its new spearheading role in stimulating revitalization and extending its services beyond the grounds of the three institutions. Initially, these institutions did not coordinate as a team. Instead, they looked solely at their own piece of the neighborhood. Over the past 10 years, Midtown Inc. underwent a paradigm shift from being growth-oriented to incorporating community services. As an interviewee from the Detroit Revitalization Fellowship Program noted, WSU had a long history of neglecting the neighborhoods around them and even causing hurt to some neighborhoods as the university wanted to demolish an adjacent neighborhood for its expansion. This growth-oriented paradigm changed when the anchor institutions began to understand that the decline of Detroit was affecting them too. This change in attitude may be connected to the changes that occurred in Midtown from 2000 to 2010: the area experienced a population decline of 11.3 % and an almost 20 % increase in the number of vacant homes.

Today, the three large anchor institutions are more concerned with the role they play in Midtown. Improving the local quality of life by reducing crime and dealing with housing abandonment and vacant lots are now primary goals of Midtown Inc. One way in which safety has improved is by extending the remit of the Wayne State University Campus Police to include all of Midtown. This fits within the changing paradigm shift mentioned above; rather than just looking out for their own territory, they see the value in good security for the entire neighborhood (and even some adjacent ones). Another has been the creation of a security council consisting of WSU police, residents, and business owners. The patrolling of WSU police in Midtown neighborhoods along with other security measures has had a massive influence on neighborhood regeneration largely because Midtown began to be perceived as safe.

All of the interviewees we spoke with about Midtown were positive about the role of the WSU police in keeping the neighborhood safe. The presence of WSU community police attracted new residents, especially whites (see Table 2), as our respondents noted that the WSU community police are more responsive than the Detroit Police department. As a real estate agent put it, ‘people don’t call 911 anymore, they call WSU police.’ A new resident explained that when the public police services declined in the city, this area actually improved at the same time due to the increased presence of the WSU police. One resident stated: ‘Their response time is tremendous; we called the WSU police and they were here in 40 seconds’.

**Table 2:** Selected statistics from the four neighborhoods and the city of Detroit

	Population			Number of households			Number of vacant homes			Median age
	2000	2010	% change	2000	2010	% change	2000	2010	% change	2010
Downtown	5373	5302	−1.3	2123	3308	55.8	1022	1198	17.2	42.0
Midtown	14,446	12,814	−11.3	7300	7068	−3.2	2004	2402	19.9	34.6
Woodbridge	3111	2942	−5.4	1314	1264	−3.8	1571	1501	−4.5	35.6
Corktown	1253	1192	−4.9	539	572	6.1	112	197	75.9	37.0
City of Detroit	951,270	713,777	−25	336,428	269,445	−19.9	38,668	79,725	106.2	34.8

	Black			White			Hispanic		
	2000	2010	% change	2000	2010	% change	2000	2010	% change
Population by race									
Downtown	4518	3337	−26.1	1290	1399	8.4	124	167	34.7
Midtown	11784	9117	−22.6	3133	3488	11.3	321	310	−3.4
Woodbridge	2308	1930	−16.4	634	787	24.1	60	98	63.3
Corktown	435	487	12.0	475	448	−5.7	305	206	−32.5
City of Detroit	775,729	590,226	−23.9	116,672	75,758	−35.1	47,168	48,679	3.2

*Source* US Census 2000, 2010

Midtown Inc has taken a leadership role in providing other services and amenities within this part of Detroit. This includes the creation of bicycle lanes—a task normally reserved for a city’s transportation department. As a Midtown Inc representative noted, “someone has to act as the leading organization to speak, lead, direct, guide, to create vision and implement and coordinate all the moving parts of the revitalization”. The same interviewee argued that waiting for the local government to take action would take too long because the local government has neither the resources nor the local knowledge of the neighborhood. As with the public–private partnerships surrounding the redevelopment of Campus Martius in Downtown Detroit, questions arise about accountability; input from the community is often limited or selected.

Midtown Inc., through its brand ‘Live Midtown,’ has also been an active agent in encouraging new residential activity. The Live Midtown scheme offers employees of the three large institutions up to $20,000 cash for the purchase of a property within Midtown (www.livemidtown.com). Incentives to renovate are available for existing homeowners and tenants can also receive financial assistance towards rent. As a result, vacancy rates in Midtown are very low, despite numerous home abandonments over the past decade and gentrification is now pricing out many low-income residents. Nevertheless Midtown Inc. aims to manage gentrification. As an employee of Midtown Inc. notes: “I think gentrification can be managed or controlled by organization. First of all, Midtown has 30 % of the rentals available that are subsidized while the surrounding neighborhoods have only 6–8 % and that 30 % in Midtown will continue to be 30 %”.

### Woodbridge

**Fig. 4:**
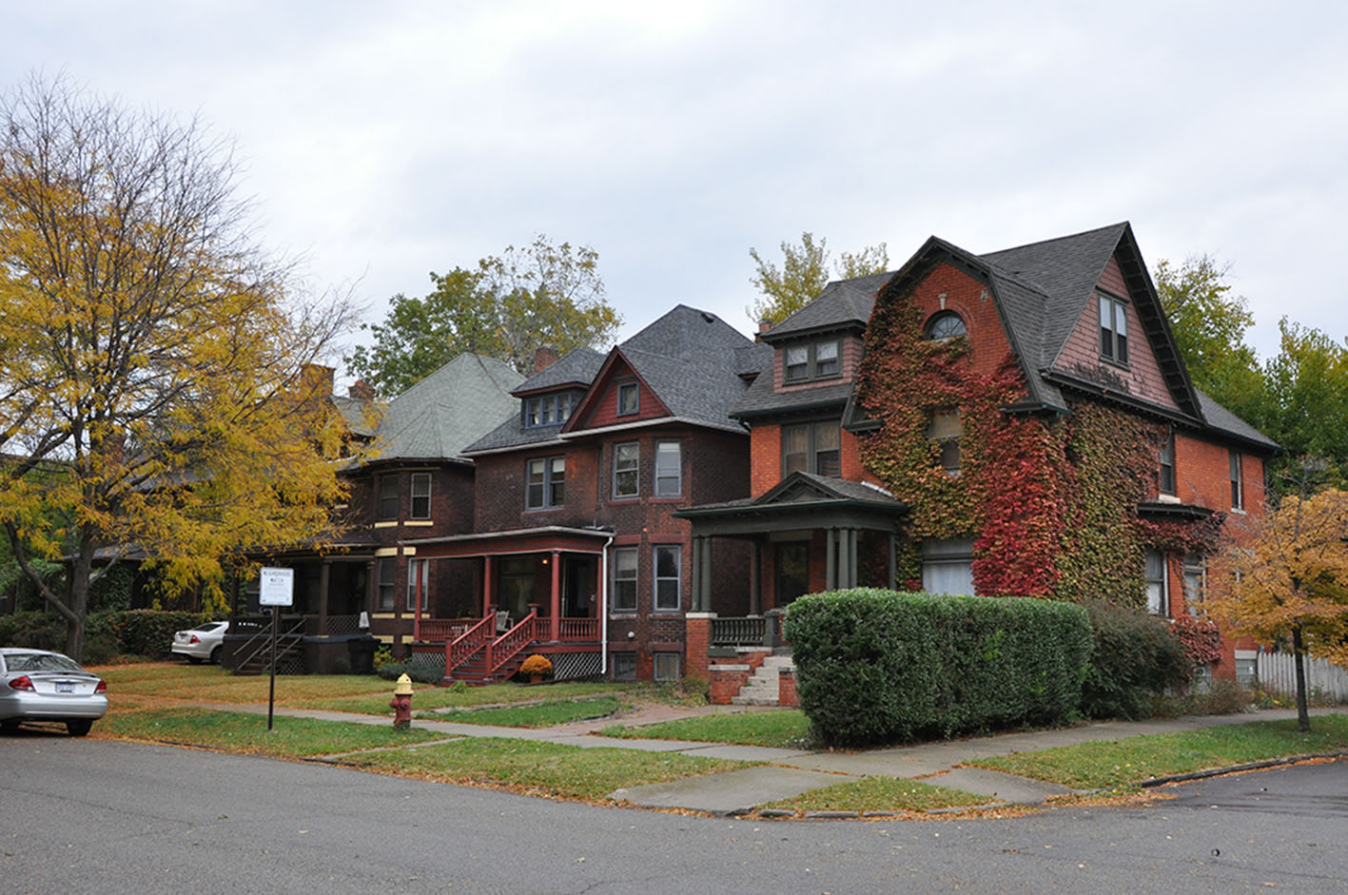
Avery Street in gentrifying Woodbridge

Woodbridge is a small residential areas situated to the west of Midtown, across the John C Lodge Freeway from Wayne State University (Fig. 4). Because of this proximity, and because Woodbridge is home to many students and faculty, WSU campus police have extended their patrols here. Consequently, Woodbridge is now far safer, and, equally important, is perceived as being a safe island in an unsafe city. As the respected Detroit-based artist Lowell Boileau explained: “Detroit is like a city of islands; if you know where they are, they are the safest places on earth.”^4^ Woodbridge is one such island.

Before the WSU police patrols began, Woodbridge was in decline. As an interviewee explained: ‘Woodbridge, in particular the areas south and west, declined heavily and had a lot of crime and fires.’ Today this has radically changed, as another of our respondents noted: ‘now there are kids on bikes without parents. You would not see that in many other neighborhoods in Detroit.’

However the regeneration in Woodbridge is highly localized and primarily concentrated around two north–south streets, Avery and Commonwealth. Surrounding these streets are large areas of blight and abandonment. Woodbridge initially tried a CDC approach but it had become inactive as a result of underfunding. Interviewees suggested that a strong community network of long-term residents and new residents exists, but that they lack scale, coordination and resources. One of the residents explained: ‘There are a lot of things where people want to participate and contribute, but people have their own lives… You really need paid staff and input from the residents rather than just sitting around talking and never getting things done.’ Woodbridge, despite its gentrifying nature, was simply too small to organize itself at the level where it could seriously impact quality of life using a CDC model. The main enhancements to quality of life have come through services from Midtown being extended into the streets of Woodbridge.

From our interviews, one other strategy employed by local residents was noted. To counter the lack of street lighting [forty percent of the streetlights in Detroit do not work (DFC 2012, p. 160)], residents adopted small-scale ‘DIY solutions’ (Kinder 2014) such as consciously leaving their porch lights on to provide at least some lighting on the streets. As one resident explained

[the darkness of unlit streets] makes it very easy for someone to commit a personal crime without being caught and it discourages people from walking at night. There were a couple of initiatives by people to put more lights on their porches to basically light up the streets since the street lights are not working

Other, localized solutions residents have employed include: organized clean-up activities (such as cleaning up dump tires, trash and maintaining abandoned buildings) and organized neighborhood watches. Residents also undertake actions to prevent neighborhood decline, for example, a resident explained that:

there was an absentee landlord on Commonwealth who let the house fall to ruin but kept paying the taxes, so it stayed in his hands, and the neighbors really pressured them to keep up the house by saying they will go to the media with pictures of the house and say that he lives in Grosse Pointe or whatever and look at what you are doing to our neighborhood.

Another example of ‘DIY urbanism’ (see Kinder 2014) involved a combination of clean-up and security patrols: ‘lawnmower patrols whereby volunteers would cut grass all season long because that really does make a difference in terms of perceptions, that somebody is taking care and watching the area’. While this indicates that residents would like to actively improve their neighborhood, it must also be acknowledged that there is a limit to what residents can do since, as our interviewee explained, “people are busy mowing their own lawns and having their own lives, so it is hard to do extra”. This highlights the contrast between this neighborhood and the other areas we studied. Because Woodbridge lacks the presence of both a strong local government and a strong private or institutional employer, it has to rely on the community and the goodwill of external institutions such as Midtown Inc and the patrols of WSU campus police. The provision of services, especially policing, comes from these external sources; Woodbridge’s community, while middle-class, is too small to provide services for itself because of the lack of resources (e.g. time, money, organization etc.). This lack of resources is common for many residential neighborhoods across Detroit (Kinder 2014) and in declining cities more generally. Woodbridge benefits from its geography and proximity; many other neighborhoods outside of The 7.2 face a much grimmer future.

### Corktown

Corktown is a small mixed-use neighborhood and a recognized historic district. It has a tight community of long-term residents and has been historically more stable than other Detroit neighborhoods. Small-scale gentrification has been occurring for several decades (Hartigan 1999). Despite this, there are many long-term residents. A local resident who we interviewed noted that, ‘houses were passed along through the family so generations grew up here and that might have helped keeping a tight community.’ Because of its proximity to Downtown and Midtown and good quality, historic homes, it has become an attractive place for professionals and artists to live in. Also the built environment plays an important role, as a mortgage broker noted: “the homes are very close together and because they have stayed intact, there is not a lot of blight in these neighborhoods. That makes these neighborhoods safer and more appealing to people”.

A strong community network of residents has undertaken a diverse range of neighborhood improvements including the construction of bicycle lanes, the organization of neighborhood watches, reducing blight and using vacant land as community space. This, and other factors, such as its housing stock and proximity to Downtown, has caused the neighborhood to grow in popularity. There are many signs of gentrification, and while this has been seen for some time, the process has been accelerating since around 2010, in parallel with the other parts of Greater Downtown. As a resident noted regarding the changes in the neighborhood: “this neighborhood is becoming very popular for hipster, white, young urban professionals to live. We now even have bike lanes, which were not here a couple of years ago…also a lot of new hip restaurants and bars are opening up, even eco-friendly stores like Green Safe Store”.

Corktown is best known for its activities along the main thoroughfare, Michigan Avenue. The corner of Michigan and Trumbull was the location of Tiger Stadium, the former home of Detroit’s namesake baseball team; game days attracted large crowds which helped support local businesses, including a large number of sports bars. The stadium closed in 1999 and was demolished a decade later. Rather than closing up, these bars have in fact thrived since the Tigers moved Downtown; many of the bars purchased second-hand school buses which shuttle fans for free to and from the game. While Detroit city buses run along Michigan Avenue towards Downtown, pub goers prefer to use these shuttles, rather than drive or use a city bus between the bars and the stadium.

**Fig. 5:**
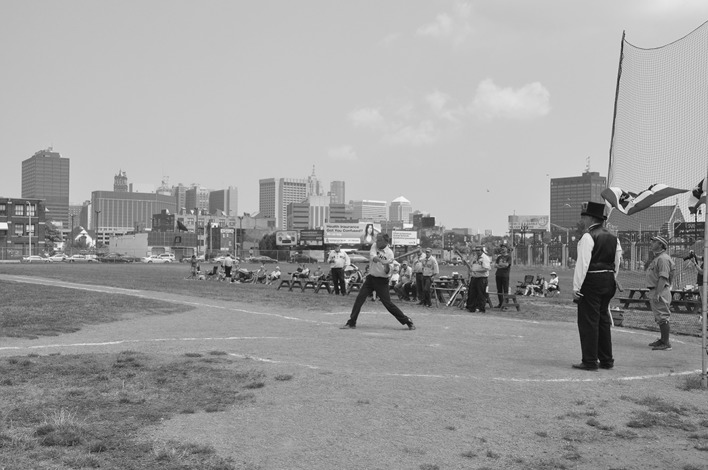
The old Tiger Stadium in Corktown, which, thanks to the Navin Field Grounds Crew, has been reimagined as a park

The old Tiger Stadium has also undergone a remarkable transformation. After several years lying vacant and abandoned, a small group of volunteers called the Navin Field Grounds Crew (Navin Field was an earlier name for Tiger Stadium) began cleaning up the old field, cutting the weeds and restoring it to a useable condition. Their activities fall within what Andrew Herscher (2012) has called ‘un-realestate’—where the absence of formal circuits of capital leads to opportunities for non-commercial and non-market activities to thrive. The site of the old Tiger Stadium is owned by the City of Detroit and legally, the Grounds Crew are trespassing when they enter the lot. But through their actions, they have created what is essentially a new park in Detroit; every Sunday, people gather to play and watch baseball (Fig. 5) (Jason Roche’s 2013 film *Stealing Home,* depicts the story of the Navin Field Grounds Crew and also highlights the tensions they faced and still continue to face with the Detroit Police).

**Fig. 6:**
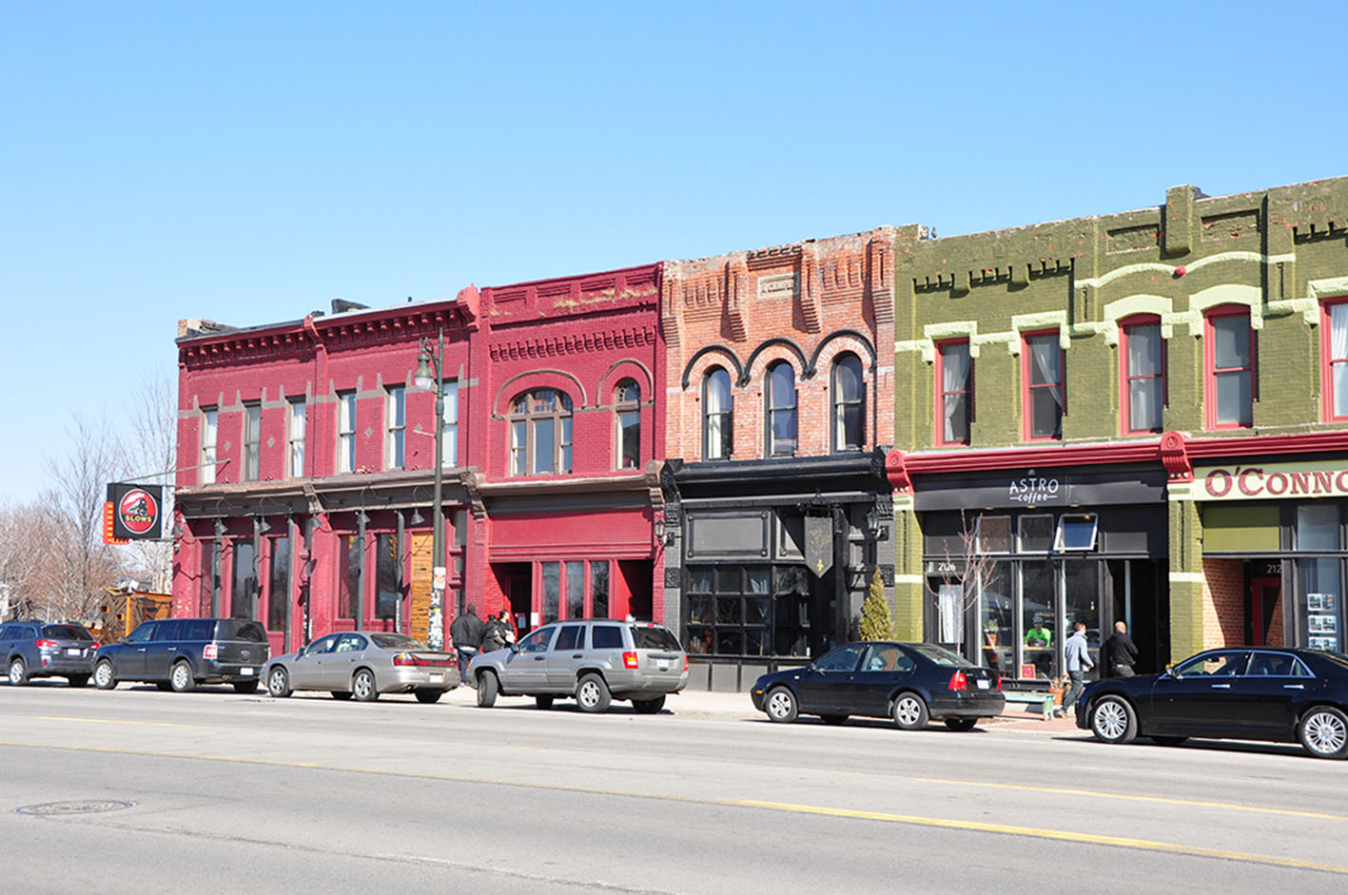
Slows BBQ (*left*) on Michigan Avenue in Corktown

Today, the main anchor of the transformation of Corktown has been a restaurant called Slows BBQ (Fig. 6). More than just for the neighborhood, Slows has become a celebrated focal point for the revitalization of Detroit. It has become the symbol of this new, trendy and hip Detroit. *The Explorer’s Guide to Detroit and Ann Arbor* (Counts 2011) writes about the importance of Slows:

While the depot [the Michigan Central Station] is the poster child for urban decay, a newer symbol of the city’s resurgence that points to the possibilities for the future is nearby on Michigan, Slows Bar-B-Q, which is jammed most days. Slows and other bars and restaurants along Michigan in the Corktown areas are a welcome sign of vitality in a city where the promise of urban renewal is long overdue (p. 129).

Counts goes on to state that: ‘this is more than a restaurant; it has become the poster child for the revitalization of downtown Detroit…Slows has become a destination for suburban residents, rather than just a place to eat before or after a sporting event, like many other downtown places’ (p. 148) Through this description, eating at Slows is about much more than eating good BBQ; it is also about consuming a piece of the revitalization of Detroit itself. Its co-owner, Phil Cooley, has been influential in the revitalization of the area surrounding his restaurant, including Roosevelt Park, situated between Slows and the Michigan Central Station.

While being one of the smallest neighborhoods in our study, Corktown shows some of the complexities and nuances in Detroit today. There are many different groups active in the neighborhood, including the Corktown Business Association, new entrepreneurs, and many local residents. But this is not without tension and controversy. Race is an important element in this equation. Most of the members of the Navin Field Grounds Crew are white and do not live in the City of Detroit (representative of the average Tigers baseball fan in general). They have asserted claim over a space they covet and hold in high regard in a city which is 85 % African American. While these differences do not play out in any violence or aggression, they show the ways in which different forces, including race, significantly affect the revitalization of Detroit. Corktown is also much whiter than the rest of Detroit; it was an Irish community and has been home to a tight-knit Maltese community for several decades. This neighborhood illustrates that a local community that consists of both private businesses and residents can organize and create services and amenities which make a difference. Compared with Woodbridge, there are more resources (including a larger business community and retail space). Compared with many other parts of the city, the historic and largely intact housing stock in Corktown gives the area comparative advantages in its economic and social capital. What the process in Corktown also shows us is that regeneration can be largely spearheaded by a small number of influential groups, businesses and individuals within the community. Yet while the spaces they create tend to be judged as ‘successful,’ they may not always reflect the needs, goals or aspirations of the wider neighborhood or the city as a whole.

## Conclusions and discussion: the 7.2 and the production of inequality

Detroit is a highly fragmented city. Greater Downtown, quantified as *Detroit 7.2*, is rapidly becoming a series of prosperous islands where investments are taking place and improvements are noticeable. As our interviews and analysis showed, business leaders, community organizations and middle-class residents in *The 7.2* are celebrating this renaissance. The new services and amenities have increased quality of life for those who live and work in Greater Downtown Detroit. The narrative of the city is even starting to shift away from decline and abandonment towards hipsters and renewal.

However, when we critically examine where this is happening and who is benefiting, it gives us pause for concern. In a city which is overwhelmingly black, much of what is taking place in Greater Downtown is being spearheaded and enjoyed by whites. *The 7.2* represents 5 % of both the city’s area and population. Much of the rest of the city is still in economic, social and demographic decline. And while emerging from bankruptcy has enabled the municipality to focus on service provision once again, this comes after years in which even basic services and amenities were lacking. In this context, different combinations of actors in the various *islands of prosperity* took it upon themselves to provide the necessities of urban life fit for capital investment and middle-class consumption. As they become more prosperous, safe and attractive places to be, this 5 % of the city will increasingly detach itself economically, racially and socially from the other 95 %. Detroit is not unique; splintering urbanism, fragmentation and privatization of urban space happens in cities around the world, furthering the boundaries between ‘core and periphery’ (Amin 1994; Soja 2000; Graham and Marvin 2001).

What our research has demonstrated is that Greater Downtown is becoming less reliant on services and amenities which would be provided, used and paid for as municipal services across the city. Business leaders and institutions have been able to provide their own police and security forces, which have reduced crime and made these areas more attract to live and invest in. Local development organizations and businesses support the creation of cycling infrastructure and other improvements to the public realm. Long-neglected public spaces have been renovated and modernized. Parks have been created by energetic and passionate volunteers. And local businesses provide shuttle transport for their patrons and employees. These and other private initiatives have led to improved quality of life and enhanced investment opportunities in the areas they serve. When only examining these areas, it is easy to understand how some will draw the conclusion that Detroit has entered a new ‘renaissance.’

But combined, these efforts contribute to a greater fragmentation of the city, which, in the long-run, will have negative consequences for the other 95 % of Detroit that is not part of this ‘renaissance.’ When examined at a city-wide level, we see that these private initiatives we have highlighted contribute to greater social and spatial inequality across the city. They all serve their immediate surroundings within Detroit’s economic and social ‘core.’ Apart from the example of WSU police patrolling nearby Woodbridge, there was very little thought as to the impact they would have on neighboring areas or the city as a whole. In short, they were locally planned and targeted towards small geographic areas. As such, they are quintessential examples of Graham and Marvin’s (2001) ‘splintering urbanism.’ In Detroit, this fragmentation manifests itself not only in spatial and class lines, but through race, as largely poor, African American neighborhoods are socially and economically distant from the city’s core.

Those living, working and investing in Greater Downtown Detroit now have less and less interest in ensuring good quality municipal services are provided throughout the city. They have opted, through their ability to fund or lead initiatives, to provide their own services and spaces instead. By doing so, they detach themselves from the fate of the rest of Detroit. In the years leading up to the bankruptcy, this was a necessity in order to attract capital and affluent residents—Detroit simply did not have the budget to provide the base services needed for mainstream American consumption. While emerging from bankruptcy means that the city has some extra money to spend on services, the quality of these are now less important to the people who live, work and play within *The 7.2* because they have already established their own private ones.

We argue that such a model actively produces fragmentation and polarization that leads to greater spatial inequality in cities such as Detroit. The most powerful and (politically) well connected parts of the city now have less need for well-funded municipal services. When billionaire Dan Gilbert is seen as the knight in shining armor for Downtown and uses his own money to refurbish Campus Martius, there is less interest there in ensuring that the city maintains and supports municipal parks. Why should Midtown residents fight for better police when they now rely on the local Wayne State University campus police? Inequality is an active process and does not just ‘happen;’ it is the product of economic, political and business decisions of what, how and where to invest and redistribute money and resources. These decisions directly contribute to the active production of—paraphrasing Berry (1985)—islands of prosperity in seas of decay. While cities have always been divided between rich and poor areas, in Detroit, these are being demarcated by the ability of private groups to fund and implement the basic necessities of a modern city.^5^

In addition to their contribution to growing social and spatial inequality across the city, such initiatives raise four important issues which are often ignored in much of the discourse on Detroit’s current renaissance:

1. Accountability: none of the examples we have cited is accountable to a democratically-elected body.
2. The privatization of space: much of Downtown, including Detroit’s main civic square, Campus Martius is now effectively planned, managed and controlled by private interests. This space is now being used to further the commercial aims of leading downtown business elites.
3. The privatization of security: in contemporary Detroit, one must now provide one’s own police force in order to provide a safe environment for capital, employees and residents. The geography of this means that most parts of the city, particularly those that need it most, do not have access to such services.
4. The gentrification of urban space: Greater Downtown Detroit is becoming ever-more gentrified and cleansed of its low-income (and to a large extent African American) residents.

**Fig. 7:**
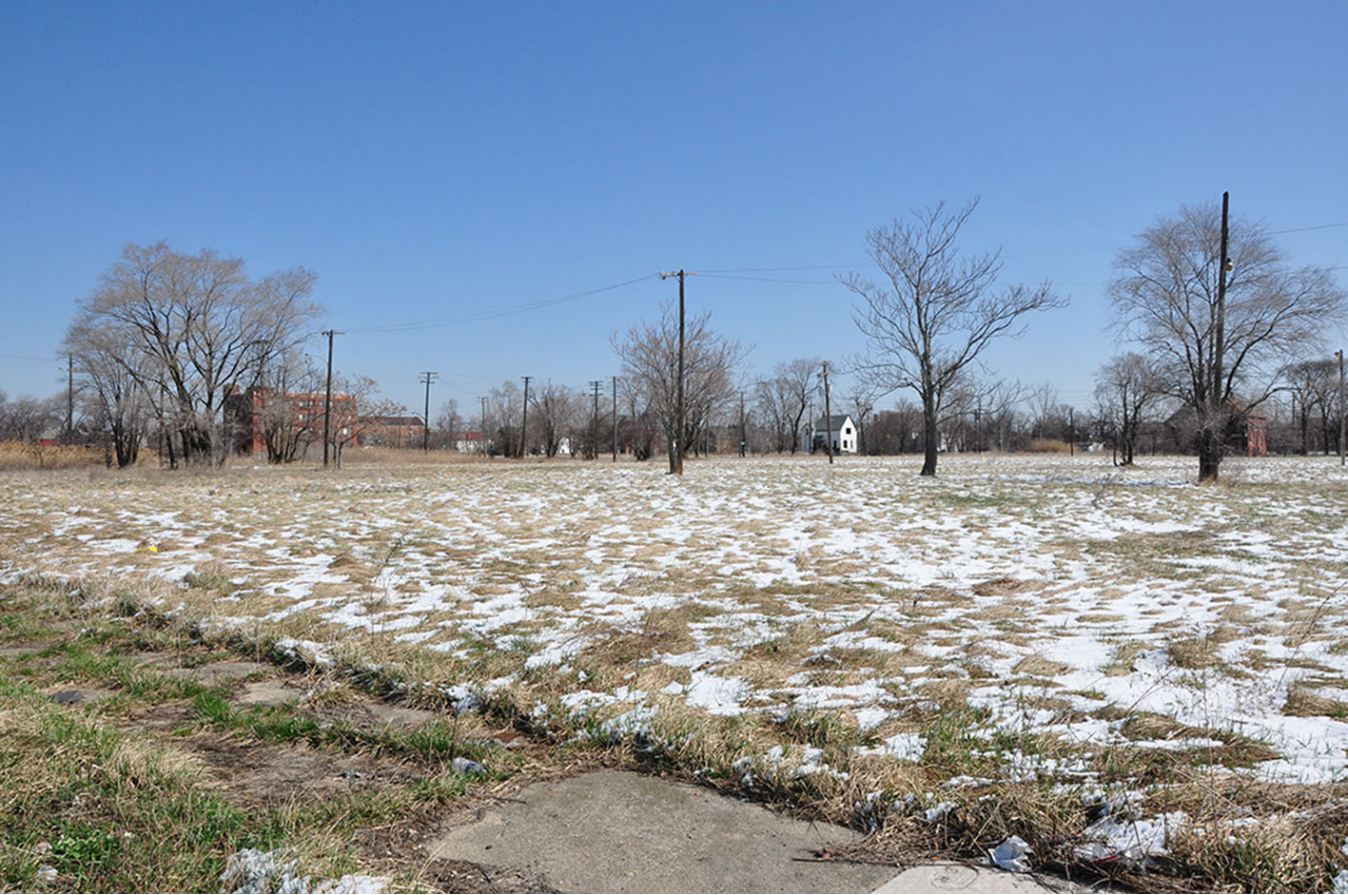
Outside of the revitalized areas we discussed, much of Detroit looks like this. This is the near East Side of Detroit, less than five minutes drive from Midtown

Detroit is being looked at with closer scrutiny than almost any other city. Therefore what happens here will not only shape the Motor City, but potentially hundreds of other cities across America and beyond. Detroit’s downtown renaissance is becoming a major point of celebration, both in the media and among some academic circles (Ager 2015; Austin 2014; Coyle 2014; Florida 2013, PPS). That is why critical urban scholarship is needed to illustrate the social and spatial consequences of the impact of what happens in Downtown Detroit beyond its borders. Far from being unique, we see Detroit as representative of fragmented, neoliberal urbanism, where a combination of residents with economic and social capital, businesses and institutions all help to produce functioning and prosperous urban islands among a sea of increased poverty, despair and political, civic and institutional neglect (see Fig. 7). In this vein, we reiterate the recent words of Akers and Leary (2014) as they comment on Dan Gilbert’s transformation of downtown: “Detroit’s atomization is inexplicably reframed as a commentary on its revitalization. And while many city residents in the (gentrifying) downtown area surely benefit from Gilbert’s infrastructure investments, can anyone plausibly argue that this is a solution for the city as a whole? … might the need to fund one’s own security and street lighting be an insurmountable barrier to all but the most wealthy and well connected?” Seen from this light, Detroit is becoming far less of an outlier among contemporary cities and more and more a poignant example of the fragmented, polarized and self-reliant urbanism of the Twenty-First Century where those with economic and social capital reap its rewards.

## Footnotes

1 see http://detroitsevenpointtwo.com/.

2
http://factfinder.census.gov/faces/tableservices/jsf/pages/productview.xhtml?src=CF.

3
http://zagster.com/detroit/.

4 Quote from the film ‘Requiem for Detroit?’ 2009 by Julien Temple.

5 It should also be noted that in the few prosperous islands outside The 7.2 (such as Palmer Park or Indian Village), private security patrols residential streets as well.
